# Impact of different guidewires on the implantation depth using the largest self-expandable TAVI device

**DOI:** 10.3389/fcvm.2022.1064916

**Published:** 2023-01-05

**Authors:** Verena Veulemans, Nihal Wilde, Hendrik Wienemann, Rik Adrichem, Thijmen W. Hokken, Baravan Al-Kassou, Jasmin Shamekhi, Victor Mauri, Oliver Maier, Christian Jung, Patrick Horn, Matti Adam, Georg Nickenig, Stephan Baldus, Nicolas M. Van Mieghem, Malte Kelm, Alexander Sedaghat, Tobias Zeus

**Affiliations:** ^1^Department of Cardiology, Pulmonology, and Vascular Diseases, University Hospital Düsseldorf, Düsseldorf, Germany; ^2^Cardiovascular Research Institute, Düsseldorf, Germany; ^3^Department of Medicine II, Heart Center, University Hospital Bonn, Bonn, Germany; ^4^Department of Cardiology, Heart Center, University of Cologne, Cologne, Germany; ^5^Department of Cardiology, Erasmus University Medical Center, Rotterdam, Netherlands

**Keywords:** TAVI, elderly, complications, implantation depth, outcome

## Abstract

**Background:**

The deployment process of the largest self-expandable device (STHV-34) during transcatheter aortic valve implantation (TAVI) might be challenging due to stabilization issues. Whether the use of different TAVI-guidewires impact the procedural success and outcome is not well-known. Therefore, we sought to evaluate the impact of non-Lunderquist (NLu) vs. the Lunderquist (Lu) guidewires during TAVI using the STHV-34 on the procedural and 30-day outcomes.

**Methods:**

The primary study endpoint was defined as the final implantation depth (ID) depending on the selected guidewire strategy. Key secondary endpoints included VARC-3-defined complications.

**Results:**

The study cohort included 398 patients of four tertiary care institutions, of whom 79.6% (317/398) had undergone TAVI using NLu and 20.4% (81/398) using Lu guidewires. Baseline characteristics did not substantially differ between NLu and Lu patients. The average ID was higher in the Lu cohort (NLu vs. Lu: −5.2 [−7.0–(−3.5)] vs. −4.5 [−6.0–(−3.0)]; *p* = 0.022^*^). The optimal ID was reached in 45.0% of patients according to former and only in 20.1% according to nowadays best practice recommendations. There was no impact of the guidewire use on the 30-day outcomes, including conduction disturbances and pacemaker need (NLu vs. Lu: 15.1 vs. 18.5%; *p* = 0.706).

**Conclusion:**

The use of the Lunderquist^TM^ guidewire was associated with a higher ID during TAVI with the STHV-34 without measurable benefits in the 30-day course concerning conduction disturbances and associated pacemaker need. Whether using different guidewires might impact the outcome in challenging anatomies should be further investigated in randomized studies under standardized conditions.

## 1. Introduction

Transcatheter aortic valve implantation (TAVI) has evolved as a standard of care for treating symptomatic severe aortic stenosis (1). In the last two decades, significant technological advancements such as retrievability, smaller sheath sizes, and new skirts reduced procedure-related complications and optimized procedural and long-term outcomes (2, 3). The newer-generation self-expandable 34 mm Evolut^TM^ R valve (Medtronic, Minneapolis, MN, USA; STHV-34) extended the annulus diameter range up to 30 mm, thus allowing the coverage of large annulus sizes (4, 5). However, the deployment process of the largest device might be challenging due to stabilization issues, leading to a prolonged procedure time and suboptimal implantation depth (ID) in specific anatomies. Growing evidence suggests an optimal ID to avoid conduction disturbances and to reach the implanted valve's best functional integrity (6–8). TAVI-guidewires guide the device through the iliofemoral arteries and aorta, similarly stretching out tortuosities, providing support during the native valve crossing, preventing nose cone injury and ventricular perforation. The following three guidewires are predominantly used in STHV-34 procedures depending on the institutions' preference and wire experience to guarantee the most appropriate stabilization during valve deployment: Safari2^TM^ (Boston Scientific, Marlborough, MA, USA), Confida^TM^ Brecker (Medtronic, Minneapolis, MN, USA), and the Lunderquist^TM^ (Cook medical, Inc., Bloomington, IN). However, their profiles concerning stiffness and stabilization ability substantially differ and may influence the implantation process to a greater extent in large anatomies. From a technical perspective, the Confida^TM^ Brecker is similar to the Safari2^TM^, both offering favorable shape retention and facilitating a stable and atraumatic valve deployment through “mid-weight” stiffness. The Lunderquist^TM^ is one of the stiffest guidewires and is available in a double-curved form. Even though little literature exists about tools to optimize ID during valve deployment (9–11), the impact of different guidewires is yet anecdotal, and structured data are still missing. Thus, we hypothesized that the stiffer Lunderquist guidewire would be superior to other guidewires and would enhance stabilization during STHV-34 deployment, assuring a higher and more controlled implant depth that might translate into higher procedural success and lower permanent pacemaker implantation (PPI) rates.

## 2. Material and methods

### 2.1. Study population

**Figure 1:**
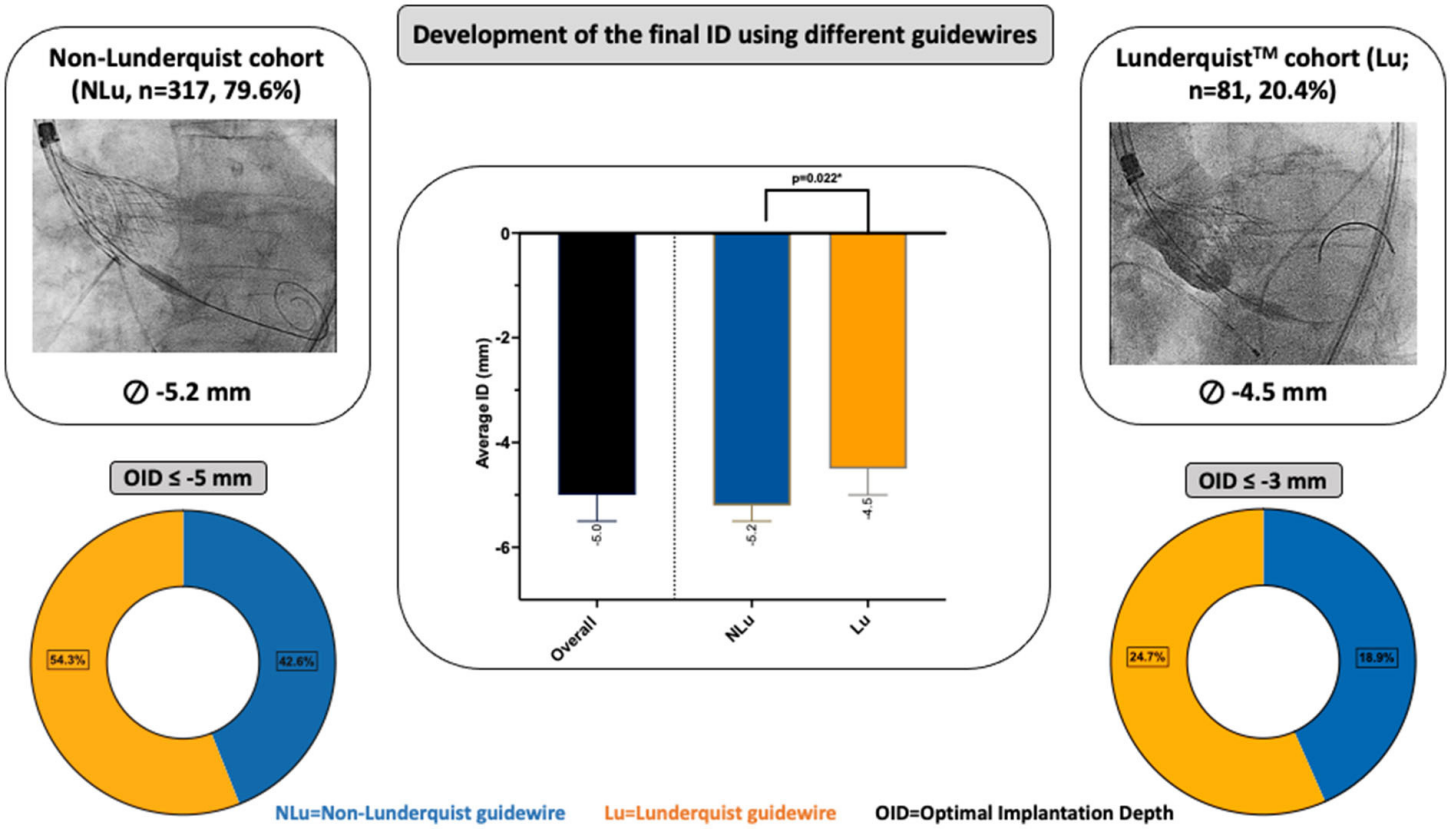
Central Illustration about study layout and results. Development of the final implantation depth (ID) using different guidewires and their influence on the optimal ID (OID).

We retrospectively enrolled 398 patients with severe AS who underwent transcatheter aortic valve implantation (TAVI) with the STHV-34 device valve (Medtronic, Minneapolis, MN, USA) between 2017 and 2021 in four tertiary care institutions. Patients with a degenerated surgical aortic bio prosthesis, pure aortic regurgitation, and suboptimal imaging studies were excluded from this analysis. All patients provided written informed consent to use clinical, procedural, and follow-up data for research. The study was conducted in accordance with the declaration of Helsinki and did not fall under the scope of the Medical Research Involving Human Subjects Act per Institutional Review Boards' review (MEC-2021-0349). According to the guidewire properties, the initial study cohort was stratified according to guidewire use into a non-Lunderquist guidewire (Safari/Confida; NLu; *n* = 317; 79.6%) cohort and a Lunderquist guidewire (Lu; *n* = 81; 20.4%) cohort. As the patient characteristics of these cohorts did not substantially differ, no propensity matching was performed with respect to the patient numbers. A full overview of the study design and the most important read-outs are displayed in Figure 1.

### 2.2. Procedural details and 3D image analysis of MSCT

The final device ID was measured by aortography in a co-planar view with the three native cusps aligned, assuring a coaxial frame position. In detail, the final ID was measured from the edge of the frame up to the nadir of the non-coronary cusp (NCC) and left coronary cusp (LCC). Valve oversizing was calculated as (prosthesis size – native annulus size/native annulus size) x100.

Fast pacing (FP) was defined as an episode of ventricular pacing between 100 and 160 bpm to reach a systolic blood pressure <100 mmHg during the final valve release (small pressure amplitude). Rapid pacing (RP) was defined as an episode of ventricular pacing between 180 and 200 bpm with the goal of inhibiting cardiac output during the final valve release. Fast and rapid pacing were either realized through a temporary pacemaker device using a transfemoral approach or a temporary guidewire-pacing.

Contrast-enhanced cardiac MSCT-studies were performed prior to the TAVI-procedure. Imaging included an ECG-gated contrast enhanced scan with multiple phases reconstructed during systole (at every 5% between 20 and 50% of the R-R interval). MSCT was analyzed by 3mensio structural heart package (Pie Medical Imaging, Maastricht, the Netherlands). The aortic valve and root were automatically reconstructed from the ECG-gated contrast scan. Dimensions were determined with the use of workstation tools. A tubular configuration of the aortic root (“tube”) was considered, when the mean aortic annulus and LVOT diameter matched in size toward a ratio of 0.9–1.1. A flared configuration was considered when the mean LVOT diameter was smaller than the mean annulus diameter (ratio >1.1). A tapered configuration (mean diameter of the LVOT greater than the mean annulus diameter) fulfilled the ratio <0.9. Calcium volume and the resulting Agatston score was determined for the aortic valve region and the LVOT as previously described.

### 2.3. Study endpoints

The primary study endpoint was defined as the final ID depending on the selected guidewire strategy. Secondary endpoints were defined as the impact of the guidewire strategy on thirty-day outcomes according to the VARC-3 definitions (12).

### 2.4. Statistical analysis

Distribution of continuous variables were tested for normality with the Shapiro-Wilk test. Continuous variables were reported as mean ± standard deviation or median (interquartile range) and analyzed with a student's *T*-test, ANOVA, Mann Whitney U- or Kruskal-Wallis-test as appropriate. Categorical variables were reported as percentage and compared with Chi-Square or Fishers Exact test. A two-sided *p*-value <0.05 was considered statistically significant. All statistics were performed with SPSS-software version 28.0 (SPSS, Chicago IL, United States).

## 3. Results

### 3.1. Baseline characteristics

Baseline characteristics did not substantially differ between Non-Lunderquist (NLu) and Lunderquist (Lu) patients. However, NLu patients had a lower STS-Score (NLu vs. Lu: 2.9 [1.9–4.8] vs. 3.5 [1.9–7.7]; *p* < 0.001^*^). A full overview of the baseline clinical and functional characteristics is displayed in Supplementary Table 1.

### 3.2. General procedural characteristics

**Figure 2:**
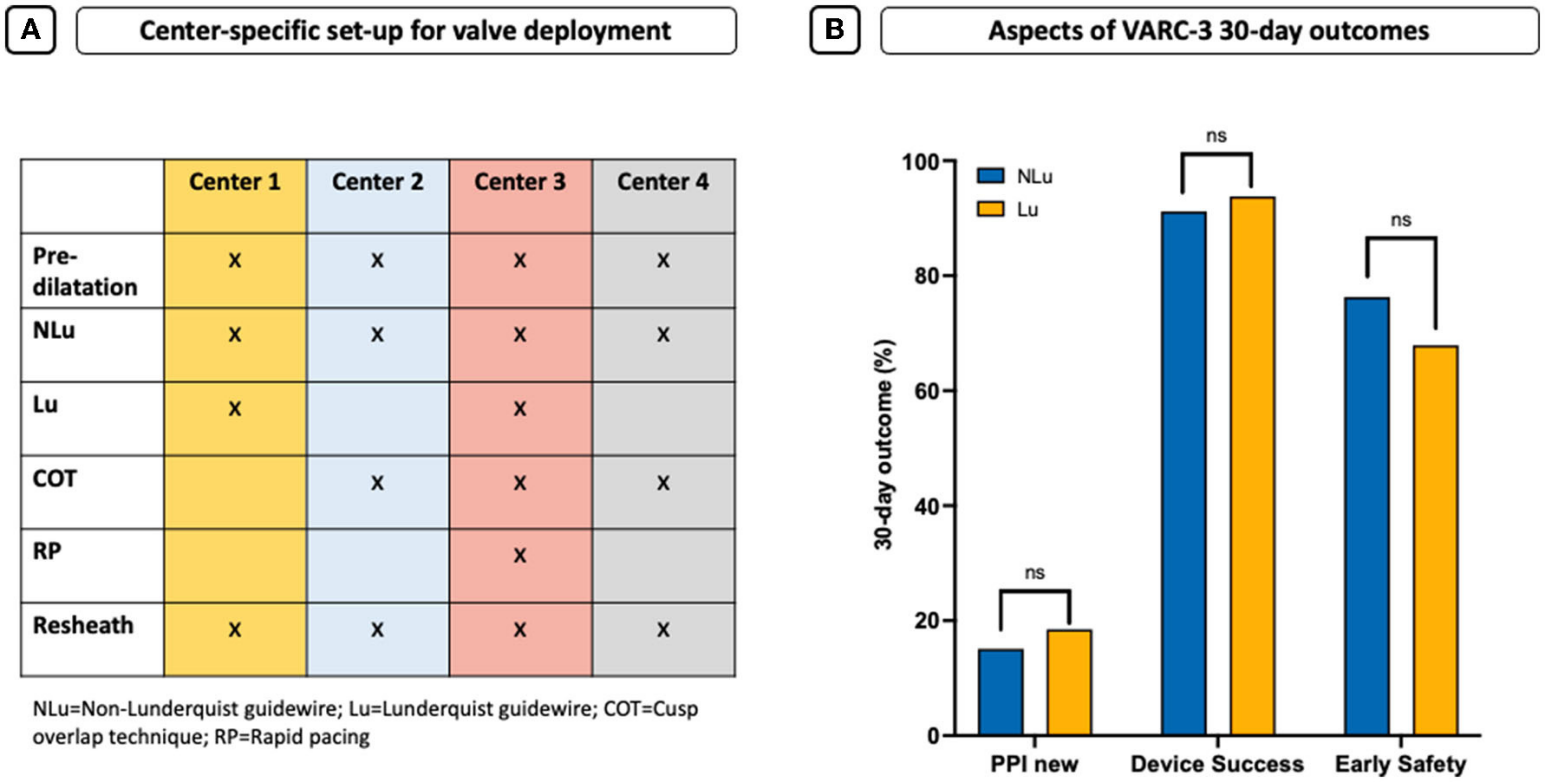
Center-specific set-up for valve deployment and 30-day outcome. **(A)** Center-specific deployment characteristics that might contribute to different IDs. **(B)** Aspects of VARC-3 30-day outcomes shown as new pacemaker need (PPI), early device success, and early safety.

Procedural details and clinical outcomes are displayed in Table 1. The transfemoral access was performed in 97.5% of all cases. The Evolut R^TM^ was the default device using the STHV-34 (88.5%), followed by the Evolut Pro^TM^ device (10.6%). Regarding the NLu cohort, 76.7% of procedures were performed using the Confida^TM^ and 23.3% using the Safari2^TM^ guidewire. Contrast use (NLu vs. Lu: 89.0 ml [70.0–116.0] vs. 80.0 ml [65.0–96.2]; *p* = 0.001^*^) was lower in the Lu cohort, although previous repositioning maneuvers were more common (NLu vs. Lu: 30.3 vs. 48.2%; *p* = 0.005^*^). The cusp overlap technique (COT) was only used in 32.4% of all patients, and less frequently in the NLu cohort (NLu vs. Lu: 21.1 vs. 76.5%; *p* < 0.001^*^). Rapid pacing (RP) was realized in 35.2% of the overall population and predominantly in Lu patients (NLu vs. Lu: 30.6 vs. 53.1%; *p* < 0.001^*^). The average implantation depth (ID) was higher in the Lu cohort (NLu vs. Lu: −5.2 mm [−7.0–(−3.5)] vs. −4.5 mm [−6.0–(−3.0)]; *p* = 0.022^*^), whereas the number of all optimal IDs (OID) above −5mm according to former recommendations was only higher by trend (NLu vs. Lu: 42.6 vs. 54.3%; *p* = 0.058^*^). Regarding updated recommendations with a target high of – 3 mm, the OID was only reached in 18.9% of NLu and 24.7% of Lu patients (*p* = 0.248). All intraprocedural complications were comparable between both cohorts. Figure 2A shows the several center-specific deployment characteristics that might contribute to different IDs in this context.

**Table 1:** Patient procedural characteristics.

**Clinical data**	**Overall** **(*n* = 398; 100%)**	**Non-Lunderquist** **(*n* = 317; 79.6%)**	**Lunderquist^*TM*^** **(*n* = 81; 20.4%)**	***p*-value**
TF access	388 (97.5)	308 (97.2)	80 (98.8)	0.652
Evolut R^TM^	354 (88.5)	287 (90.5)	67 (82.7)	0.088
Evolut Pro^TM^	42 (10.6)	28 (8.8)	14 (17.3)	0.054
Evolut Pro+^TM^	2 (0.5)	2 (0.6)	0 (0.0)	0.723
Confida^TM^	243 (61.1)	243 (76.7)	0 (0.0)	< 0.001*
Safari^2TM^	74 (18.6)	74 (23.3)	0 (0.0)	< 0.001*
Contrast, ml	85.0 [70.0–110.0]	89.0 [70.0–116.0]	80.0 [65.0–96.2]	0.001*
Fluoroscopy time, min	20.0 ± 9.6	19.5 ± 9.0	21.8 ± 11.5	0.104
Pre-dilatation	174 (43.7)	138 (43.5)	36 (44.4)	0.986
Post-dilatation	76 (19.1)	59 (18.6)	17 (21.0)	0.861
COT projection	129 (32.4)	67 (21.1)	62 (76.5)	< 0.001*
Rapid pacing	140 (35.2)	97 (30.6)	43 (53.1)	< 0.001*
Resheath/-capture	135 (33.9)	96 (30.3)	39 (48.2)	0.005*
Repetitive resheath/-capture	41 (10.3)	27 (8.5)	14 (17.3)	0.041*
ID (average mean)	−5.0 [−7.0–(−3.5)]	−5.2 [−7.0–(−3.5)]	−4.5 [−6.0–(−3.0)]	0.022*
ID ( → NCC)	−4.0 [−6.0–(−3.0)]	−4.0 [−6.0–(−3.0)]	−4.0 [−5.5–(−2.0)]	0.009*
ID ( → LCC)	−6.0 [−8.0–(−4.0)]	−6.0 [−8.0–(−4.0)]	−6.0 [−7.0–(−3.8)]	0.085
OID ≤ −5 mm ab annulus	179 (45.0)	135 (42.6)	44 (54.3)	0.058
OID ≤ −3 mm ab annulus	80 (20.1)	60 (18.9)	20 (24.7)	0.248
**Intraprocedural complications**				
Immediate stroke	2 (0.5)	1 (0.3)	1 (1.2)	0.505
Aortic dissection	0 (0)	0 (0)	0 (0)	1.000
Annulus rupture	0 (0)	0 (0)	0 (0)	1.000
Coronary obstruction	1 (0.3)	0 (0)	1 (1.2)	0.093
Vascular complications	19 (4.8)	14 (4.4)	5 (6.2)	0.758
Valve dislocation	2 (0.5)	2. (0.6)	0 (0)	0.723
Conversion to surgery	0 (0)	0 (0)	0 (0)	1.000
Need of 2nd valve	4 (1.0)	3 (1.0)	1 (1.2)	0.966
Tamponade	1 (0.3)	1 (0.3)	0 (0)	0.850
CPR	2 (0.5)	1 (0.3)	1 (1.2)	0.505

Values are mean ± SD, median ± interquartile range or n (%). ^*^p-value < 0.05.

COT, cusp overlap technique; CPR, cardiopulmonary resuscitation; (O)ID, (optimal) implantation depth; TF, transfemoral.

Thus, a subanalysis in 202 patients acknowledging the reached ID without the use of COT or RP in the several cohorts (NLu vs. Lu: *n* = 185 vs. *n* = 17) revealed again that the average ID was higher in the Lu cohort (NLu vs. Lu: −5.5 mm [−7.5–(−4.0)] vs. −4.1 mm [−5.0–(−4.0)]; *p* = 0.031^*^), also including the number of all OID above −5 mm according to former recommendations (NLu vs. Lu: 35.7 vs. 70.6%; *p* < 0.001^*^; Supplementary Table 2).

### 3.3. 30-day outcome and functional status

All 30-day outcome characteristics were comparable (Table 2). In particular, the amount of conduction disturbances and the need for permanent pacemaker implantation (PPI) were similar and high in both cohorts (NLu vs. Lu: 15.1 vs. 18.5%; *p* = 0.706, Figure 2B).

**Table 2:** 30-day outcome according to VARC-3.

**Clinical data**	**Overall** **(*n* = 398; 100%)**	**Non-Lunderquist** **(*n* = 317; 79.6%)**	**Lunderquist^*TM*^** **(*n* = 81; 20.4%)**	***p*-value**
30-day mortality	4 (1.0)	3 (1.0)	1 (1.2)	0.966
Peri- and post-procedural MI	0 (0)	0 (0)	0 (0)	1.000
**All CVE**	13 (3.3)	9 (2.8)	4 (4.9)	0.568
NeuroARC 1	4 (1.0)	3 (1.0)	1 (1.2)	0.966
TIA	7 (1.8)	6 (1.9)	1 (1.2)	0.902
**Bleeding**	82 (20.6)	64 (20.2)	18 (22.2)	0.902
Type I	51 (12.8)	40 (12.6)	11 (13.6)	0.920
Type 2	23 (5.8)	16 (5.0)	7 (8.6)	0.526
Type 3	7 (1.8)	6 (1.9)	1 (1.2)	0.816
Type 4	2 (0.5)	2 (0.6)	29 (3.6)	0.681
**VASC**	83 (20.9)	67 (21.1)	16 (19.8)	0.954
Major VASC	21 (5.3)	18 (5.7)	3 (3.7)	0.728
Minor VASC	62 (15.6)	49 (15.5)	13 (16.1)	0.989
Closure device failure	10 (2.5)	8 (2.5)	2 (2.5)	1.000
**AKI I-IV**	48 (12.1)	37 (11.7)	11 (13.6)	0.869
AKI I	39 (9.8)	29 (9.2)	10 (12.4)	0.625
AKI II	3 (0.8)	3 (1.0)	0 (0)	0.615
AKI III	3 (0.8)	2 (0.6)	1 (1.2)	0.819
AKI IV (new RRT)	3 (0.8)	3 (1.0)	0 (0)	0.615
New AVB (I–III°)	74 (18.6)	54 (17.0)	20 (24.7)	0.106
New LBBB/RBBB	84 (21.1)	67 (21.1)	17 (21.0)	0.999
New PPI	63 (15.8)	48 (15.1)	15 (18.5)	0.706
TAVI-related reintervention	2 (0.5)	1 (0.3)	1 (1.2)	0.505
Cardiac structural complications	3 (0.8)	2 (0.6)	1 (1.2)	0.819
Minor	0 (0)	0 (0)	0 (0)	1.000
Major	3 (0.8)	2 (0.6)	1 (1.2)	0.819
**Functional data**				
Vmax (m/s)	1.9 ± 0.4	1.8 ± 0.4	2.0 ± 0.5	0.065
dPmean (mmHg)	6.9 ± 4.3	7.0 ± 4.5	6.7 ± 3.6	0.803
EOAi (cm^2^/m^2^)	1.0 ± 0.3	1.0 ± 0.3	1.0 ± 0.3	0.893
DVI	0.6 ± 0.2	0.6 ± 0.2	0.6 ± 0.2	0.999
PVL ≥II°	16 (4.0)	14 (4.4)	2 (2.5)	0.670
Device success	365 (91.7)	289 (91.2)	76 (93.8)	0.685
Early safety	297 (74.6)	242 (76.3)	55 (67.9)	0.224

Values are mean ± SD or n (%). ^*^p-value < 0.05.

AKI, acute kidney injury; AVB, atrioventricular block; CVE, cerebrovascular events; DVI, doppler velocity index; EOA_i_, effective orifice area (index); LBBB, left-bundle branch block; PPI, permanent pacemaker therapy; PVL, paravalvular leakage; RBBB, right-bundle branch block; RRT, renal replacement therapy.

Functional improvement was observed in both groups without differences concerning prosthesis function and paravalvular regurgitation (PVL) as evaluated by the pre-discharge echocardiography (Table 2). Early device success was similar in both cohorts (NLu vs. Lu: 91.2 vs. 93.8%; *p* = 0.685, Figure 2B). Early safety showed also no statistically relevant difference but was formally lower by frequencies in the Lu cohort (NLu vs. Lu: 76.3 vs. 67.9%; *p* = 0.224, Figure 2B).

## 4. Discussion

The key findings of our retrospective multicenter study with a total of 398 patients undergoing TAVI with the STHV-34 are:

1. ID of TAVI with the STHV-34 was higher with the Lunderquist^TM^ guidewire.
2. The optimal ID was reached in 45.0% of patients according to former and only in 20.1% according to nowadays best practice recommendations.
3. Short-term outcome was not affected by guidewire selection.

Despite considerable advances in TAVI, some anatomical conditions are still challenging concerning a stable and hemodynamically favorable deployment of the transcatheter heart valve. Larger annuli might significantly affect procedural outcomes (13, 14). The Evolut R/PRO system is one of the most widely used next-generation devices, of which the largest available STHV-34 mm is unique as it covers a perimeter up to 94.2 mm. Even though the outcomes were consistently described as favorable (4, 5), the deployment of the largest device might be challenging due to stabilization issues in specific anatomies. Furthermore, post-procedural conduction disorders following TAVI using the STHV-34 still range between 15 and 35% (5, 8, 14). Recent literature addressed the role and importance of an optimal ID to avoid conduction disturbances and to achieve the best hemodynamic function (6–8). Taken into consideration that PVL-related device failure often occurs in larger annuli (15), the STHV-34 was optimized with a larger inflow diameter and advanced radial force, resulting in a better valve sealing capacity with less PVL (15, 16).

In this context, several pre-shaped TAVI guidewires are commercially available to support the implantation process. Each guidewire has specific stiffness, shape, and support features on different levels of the aortic root that must be considered during wire selection. While stiffer wires provide more stability, less stiff wires lower vascular damage and ventricular perforation risk. Generally, stiffer wires are used to optimize intravascular access, stretch out pronounced tortuosities, and introduce large diameter sheaths in complicating anatomies. Related to expert opinions, usage of the stiffer Lunderquist^TM^ might be superior to other wires through better stabilization properties in larger anatomies. The most widely used guidewire with the Evolut R/Pro system is the Confida^TM^ which was explicitly designed for this purpose. The Safari wire is available in three different loop sizes (extra small, small, and large). Even though the large version may be more suitable in the setting of larger ventricles, this exemplar is not regularly available in cath labs. The most frequently used Safari wire is the small one, often independently used from left ventricle configuration and size or other anatomical considerations. This led to the question if the use of different guidewires may probably influence the implantation process and the associated outcomes to a greater extent in large anatomies.

Our study is the first structured evaluation of the procedural impact of different guidewires in a large cohort of all-comer TAVI patients treated with the STHV-34. For this purpose, we tested the stiffer Lunderquist wire against two predominantly used comparable wires in stiffness grading. Even though the Lunderquist^TM^ guidewire was only used in approximately twenty percent of all cases, we could show that the average ID was almost 1 mm higher in the Lu cohort but still too deep concerning nowadays' recommendations. However, whether the higher ID was affected by the stiffer guidewire or is predominantly linked to some center-specific deployment characteristics is questionable. According to the current knowledge about the importance of an OID (6–8), recommendations for best practice implantation of the Medtronic self-expandable device have changed from a target ID between −3 and −5 mm toward −3 mm in 2020, also recommending a cusp overlap angulation technique (COT) to reach a higher ID (10, 11). Thus, it is noteworthy that most cases were performed with the knowledge of the initially deeper ID and without the COT technique. Furthermore, as one other optimization tool of ID as previously described (9), rapid pacing was only performed in 35.2% of all cases and predominantly in Lu patients, probably also contributing to the higher ID in the end. However, a subanalysis of the same cohorts undergoing TAVI without rapid pacing mode and COT revealed a significantly higher ID using the Lunderquist^TM^ guidewire even in a small sample size of 185 NLu vs. 17 Lu patients, which seems remarkable. In general, the OID <-5 mm was reached more frequently than in the overall cohort, while there was no difference in the OID according to the current best practice recommendations (<-3 mm).

There was no measurable impact on thirty-day outcomes, including the number of conduction disturbances and TAVI-related permanent pacemaker need. The PPI need was similar and high in both cohorts ranging between 15 and 19% and consistent with current literature (5, 8, 14). This might be due to the still deeper average ID in both cohorts being unfavorable in terms of an individualized approach for minimizing implantation depth according to the membranous septum (MIDAS, 6) and the recommended OID. However, it was also shown that most previously reported determinants fail to predict PPI need using the STHV-34, including membranous septum length and ID (8). Early device success using the STHV-34 was with an average of 92% favorable and similar in both wire-cohorts. Interestingly, early safety was formally lower by frequencies in the Lu cohort (76.3 vs. 67.9%) but failed statistical significance. This might be affected by slightly elevated fractions of VARC-3 bleeding, PPI need, and other factors that led to a summation effect regarding early safety in the Lu cohort.

## 5. Conclusion

The use of the Lunderquist^TM^ guidewire was associated with a higher ID during TAVI with the STHV-34 without measurable benefits in the 30-day course concerning conduction disturbances and associated pacemaker need. Whether the use of different guidewires is able to impact the outcome in challenging anatomies should be further investigated in larger and randomized studies under more standardized conditions.

## 6. Limitations

This multi-center retrospective analysis has inherent limitations. Device (guidewire, transcatheter heart valve, size) selection and implantation technique was not uniform but at the discretion of the respective operators. However, to our knowledge, this is the first study evaluating the impact of different guidewires on implantation depth in TAVI. Moreover, although the baseline characteristics were well-balanced between the two groups, we cannot exclude unmeasured confounders. Implantation depth by angiography is notoriously unreliable and may not correlate with MSCT depth assessment (17). Generally, two experienced implanters provided commitment on the final ID intra-procedurally based on the angiographic results.

## Data availability statement

The original contributions presented in the study are included in the article/Supplementary material, further inquiries can be directed to the corresponding author/s.

## Ethics statement

The study was conducted in accordance with the declaration of Helsinki and did not fall under the scope of the Medical Research Involving Human Subjects Act per Institutional Review Boards' review (MEC-2021-0349) and the Ethic Committee Heinrich-Heine-University Düsseldorf. The patients/participants provided their written informed consent to participate in this study.

## Author contributions

VV: conception and design, investigation, analysis and interpretation of data, drafting of the manuscript, project administration, and final approval of the manuscript. NW, HW, RA, TH, BA-K, JS, VM, OM, PH, MA, GN, CJ, and SB: analysis and interpretation of data, revision of the manuscript for important intellectual content, and final approval of the manuscript. NVM and MK: conception and design, validation, drafting of the manuscript, and final approval of the manuscript. AS and TZ: conception and design, investigation, drafting of the manuscript, project administration, and final approval of the manuscript. All authors contributed to the article and approved the submitted version.
