# Supplementary material for: Impact of different guidewires on the implantation depth using the largest self-expandable TAVI device
Source: Front Cardiovasc Med. 2023 Jan 5;9:1064916. doi: 10.3389/fcvm.2022.1064916 (PMC9849574; doi:10.3389/fcvm.2022.1064916)
Supplement: Supplementary file 1 [file Data_Sheet_1.docx]

Supplementary Material

# Supplementary Figures and Tables

## Supplementary Table 1: Patient Clinical and Functional Characteristics

| **Clinical data** | **Overall**  **(n=398; 100%)** | **Non-Lunderquist**  **(n=317; 79.6%)** | **Lunderquist^TM^**  **(n=81; 20.4%)** | **p-value** |
| --- | --- | --- | --- | --- |
| Age, years | 80.3 ± 6.8 | 80.3 ± 6.7 | 80.3 ± 7.1 | 0.999 |
| Gender, male | 366 (91.7) | 291 (91.8) | 75 (92.6) | 0.966 |
| BMI | 27.3 ± 5.0 | 27.0 ± 4.4 | 28.4 ± 6.9 | 0.049* |
| CAD | 281 (70.6) | 225 (71.0) | 56 (69.1) | 0.935 |
| Previous PCI | 173 (43.5) | 132 (41.6) | 41 (50.6) | 0.270 |
| Previous CABG | 54 (13.6) | 44 (13.9) | 10 (12.4) | 0.921 |
| Previous valve | 3 (0.8) | 3 (1.0) | 0 (0.0) | 0.615 |
| Previous PPI | 49 (12.3) | 41 (12.9) | 9 (9.9) | 0.703 |
| Arterial hypertension | 345 (87.7) | 277 (87.4) | 68 (84.0) | 0.660 |
| Diabetes mellitus | 121 (30.4) | 92 (29.0) | 29 (35.8) | 0.417 |
| PAD | 97 (24.4) | 81 (25.6) | 16 (19.8) | 0.479 |
| COPD | 96 (24.1) | 82 (25.9) | 14 (17.3) | 0.203 |
| Atrial fibrillation/flutter | 174 (43.7) | 141 (44.5) | 33 (40.7) | 0.793 |
| **Functional data** |  |  |  |  |
| STS, % | 3.0 [1.9-5.2] | 2.9 [1.9-4.8] | 3.5 [1.9-7.7] | <0.001* |
| LVEF, % | 51.5 ± 13.7 | 51.0 ± 13.4 | 53.1 ± 14.8 | 0.490 |
| AVA, cm^2^ | 0.76 ± 0.2 | 0.76 ± 0.2 | 0.77 ± 0.2 | 0.921 |
| dPmean, mmHg | 38.7 ± 14.6 | 39.0 ± 14.7 | 37.5 ± 14.5 | 0.653 |
| **MSCT data** |  |  |  |  |
| Bicuspid Valve | 40 (10.1) | 35 (11.0) | 5 (6.2) | 0.349 |
| AN perimeter, mm | 85.8 ± 4.2 | 85.6 ± 4.5 | 86.5 ± 2.9 | 0.176 |
| AN/LVOT-ratio | 1.0 [1.0-1.1] | 1.0 [1.0-1.1] | 1.0 [1.0-1.1] | 0.626 |
| Eccentricity index | 0.2 [0.1-0.2] | 0.2 [0.1-0.2] | 0.2 [0.2-0.2] | 0.938 |
| Oversizing | 24.4 ± 4.5 | 24.4 ± 4.8 | 24.0 ± 3.4 | 0.694 |
| Aortic Root Angulation (°) | 50.6 ± 9.2 | 50.4 ± 9.4 | 51.3 ± 8.5 | 0.709 |
| AVC, AU (total) | 2277  [1438-3694] | 2360  [1478-3816] | 1963  [1224-3208] | 0.368 |
| LVOT-Calcification | 183 (46.6) | 144 (45.4) | 39 (51.3) | 0.578 |
| Values are mean ± SD, median ± interquartile range or n (%). *p-value < 0.05  AF=atrial fibrillation; AN=annulus; AVA=aortic valve area; AVC=aortic valve calcification; BMI=body mass index; CABG=coronary artery bypass graft; CAD=coronary artery disease; CI=cardiac index; COPD=chronic obstructive pulmonary disease; dPmean/max=mean/max. transvalvular gradient; LVEF=Left ventricular ejection fraction; LVOT=Left ventricular outflow tract; PCI=percutaneous coronary intervention; PAD=peripheral artery disease; PPI=permanent pacemaker implantation; | | | | |

## Supplementary Table 2: Impact of different guidewires on the ID (without COT and RP)

| **Clinical data** | **Overall**  **(n=202; 100%)** | **Non-Lunderquist**  **(n=185; 91.6%)** | **Lunderquist^TM^**  **(n=17; 8.4%)** | **p-value** |
| --- | --- | --- | --- | --- |
| COT projection | 0 (0) | 0 (0) | 0 (0) | 1.000 |
| Rapid pacing | 0 (0) | 0 (0) | 0 (0) | 1.000 |
| ID (average mean) | -5.5 [-7.4-(-4.0)] | -5.5 [-7.5-(-4.0)] | -4.1 [-5.0-(-4.0)] | 0.031* |
| ID (🡪NCC) | -4.0 [-6.0-(-2.2)] | -4.7 [-6.0-(-3.0)] | -2.0 [-3.0-(-2.0)] | <0.001* |
| ID (🡪LCC) | -7.0 [-8.6-(-5.0)] | -7.0 [-8.6-(-5.0)] | -7.0 [-8.0-(-6.0)] | 0.639 |
| OID ≤ -5mm ab annulus | 78 (38.6) | 66 (35.7) | 12 (70.6) | 0.005* |
| OID ≤ -3mm ab annulus | 27 (13.4) | 25 (13.5) | 2 (11.8) | 0.839 |
| Values are mean ± SD, median ± interquartile range or n (%). *p-value < 0.05  COT=cusp overlap technique; CPR=cardiopulmonary resuscitation; (O)ID=(Optimal) Implantation depth; TF=transfemoral | | | | |
